# Atmosphere similarity patterns in boreal summer show an increase of persistent weather conditions connected to hydro-climatic risks

**DOI:** 10.1038/s41598-021-01808-z

**Published:** 2021-11-24

**Authors:** Peter Hoffmann, Jascha Lehmann, Bijan Fallah, Fred F. Hattermann

**Affiliations:** Potsdam-Institute for Climate Impacts Research, Climate Resilience, 14412 Potsdam, Germany

**Keywords:** Atmospheric science, Climate change

## Abstract

Recent studies have shown that hydro-climatic extremes have increased significantly in number and intensity in the last decades. In the Northern Hemisphere such events were often associated with long lasting persistent weather patterns. In 2018, hot and dry conditions prevailed for several months over Central Europe leading to record-breaking temperatures and severe harvest losses. The underlying circulation processes are still not fully understood and there is a need for improved methodologies to detect and quantify persistent weather conditions. Here, we propose a new method to detect, compare and quantify persistence through atmosphere similarity patterns by applying established image recognition methods to day to day atmospheric fields. We find that persistent weather patterns have increased in number and intensity over the last decades in Northern Hemisphere mid-latitude summer, link this to hydro-climatic risks and evaluate the extreme summers of 2010 (Russian heat wave) and of 2018 (European drought). We further evaluate the ability of climate models to reproduce long-term trend patterns of weather persistence and the result is a notable discrepancy to observed developments.

## Introduction

Recent years have shown that rapid climate change, as observed worldwide^18,47,51^, is manifested not only in the creeping rise of temperature but also in the accumulation and intensification of weather extremes^9,12,13,33,42,43^ and strong deviations of monthly, seasonal or annual values from normal conditions^7,10,37^. Understanding of the basic physical laws allows us to explain and classify certain long-term developments, such as the amplified greenhouse effect leading to rising temperatures, melting glaciers and increasing potential evaporation. However, unforeseen atmospheric phenomena are very likely, due to the complexity of the interacting climate system. For example, changes of dynamical factors, e.g. the tendency of critical circulation patterns to be more persistent (e.g. omega blocking patterns), in the Northern Hemisphere (NH) mid-latitudes can intensify the occurrence of multiple hydro-climatic extremes (heat waves, droughts, forest fires and floods) and this synchronized at different locations of the NH^30^. New extreme weather records can be reached as a consequence, e.g. if the same weather pattern remains in one location for a longer period of time than observed before^24,45^. The extreme summers in 2010 and 2018 are two prominent cases, where dynamical patterns of the atmosphere circulation played a crucial role in the extent of the extreme events^28,30,32,46^. However, the long-term climatic assessment of such events including the causing mechanisms require more complex diagnostics.

The search for suitable methods to identify persistent weather patterns has thus become a prominent research topic. In the NH mid-latitudes efforts are focusing on the role of planetary waves resonance^11,15,30^, atmospheric blocking events^6^ or certain regional circulation patterns at the continental scale^5,14,21,24^. Recent evaluation of reanalysis data strongly suggest that weather extremes increase in severity as a result of dynamical changes in the atmosphere. For example, Kornhuber et al.^29^ found a slowdown of translation speed of summer high and low pressure systems in mid-latitudes. The long persistence of a given weather situation can lead to extreme conditions (strong weather anomalies) over weeks^31^. Because of the severity of the events, it is of fundamental importance to get a better understanding of the underlying processes. However, understanding starts with detection and there is a need to find and further develop methods to capture and quantify persistent weather conditions^44^. For climate impact research, improved screening methods to classify, interpret and also communicate such climate patterns and uncertainties are essential^17^.

Based on established image recognition methods^1^, we developed a new index which quantitatively compares day-to-day similarities of regional atmospheric fields. The main difference with other approaches that identify blocking patterns^6^ is the setting. Every individual weather pattern, independent of the shape, location and time, is assigned to a measure of similarity compared to following weather patterns. Different metrics to quantify the degree of similarity are available^36^ but the use of a metric from image comparison applications is obvious to identify structural differences in images. Hydro-climatic extremes such as heat waves, droughts and floods^16^ can be examined from a different perspective, taking the similarity of weather patterns as a predictor for actual meteorological conditions near the Earth’s surface. In addition, such a predictor would also be a useful criterion to validate the outcome of climate models^35^. If they do not reproduce persistent weather patterns, which are often the origin of extreme weather conditions, they will also likely underestimate future weather extremes. This is particularly critical in climate scenario simulations: If missed or spatially dislocated, such underlying dynamical features cannot be introduced by downscaling^26^ or bias adjustment approaches^22^ afterwards.

## Results and discussions

The structural similarity^50^ of 10-day successive atmosphere images is introduced here as a new measure to study weather persistence associated with hydro-climatic extreme events (see Methods). Applying the new weather persistence index (WPI) to global atmosphere fields we want to answer the following research questions: (1) *Trends: Are there signs of long-term changes of regional weather persistence in NH summer?* (2) *Anomalies: Is there a signature of extreme years in weather persistence?* (3) *Linkage: Which relationship exists to other meteorological quantities?* (4) *Events: Is there a connection between hydro-climatic extreme events and persisting weather patterns?* Finally, we are concerned with the crucial question, (5) *whether the ability to capture persistence could be a necessary criterion for evaluating the performance of the global climate models (GCMs) simulations to adequately project future weather extremes.* Our study time period is 1981–2019 and the observed atmospheric data used are taken from the ECMWF Reanalysis 5th Generation (ERA5)^20^.

### Seasonal trends in WPI

**Figure 1:**
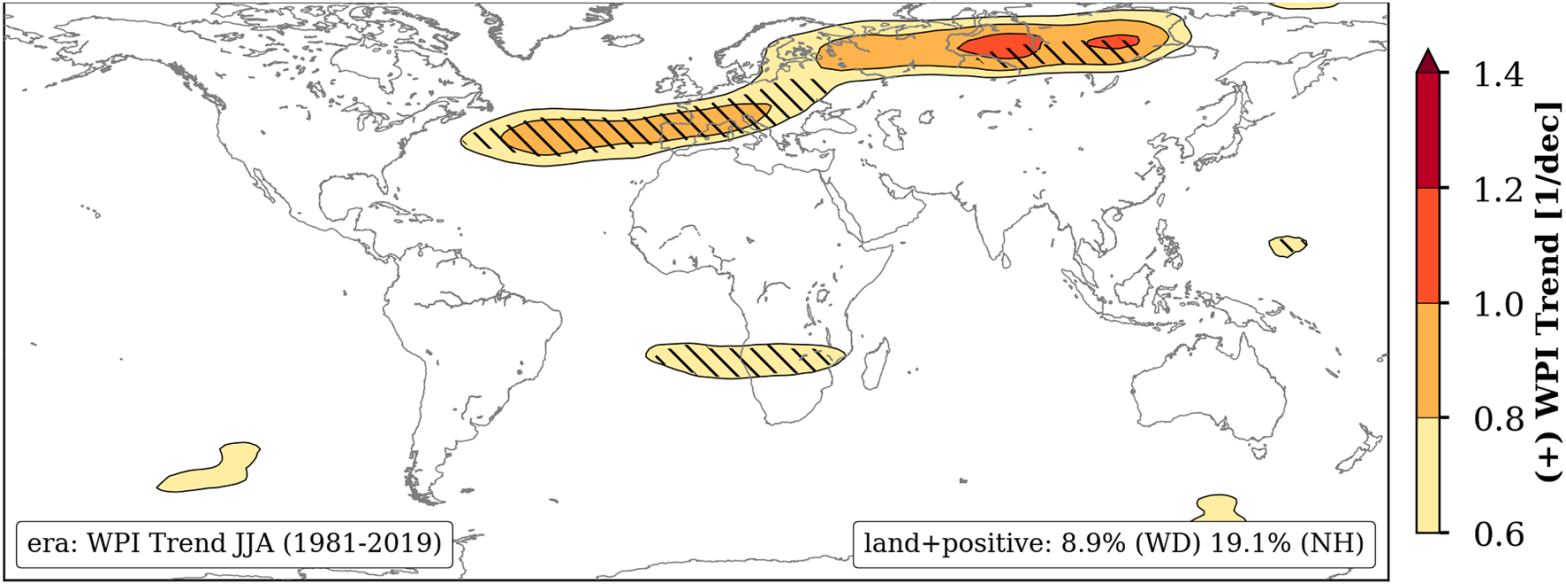
Observed trend pattern of WPI in Jun–Aug from 1981 to 2019 processed from ERA5 reanalysis data. Only positive and significant trends (*p*-value < 0.05 using a two-sided *t*-test) are shown as contours and hatched, respectively. The values in the lower right box represent the percentage of grid cells over land showing positive trends larger than $+0.6\,{\mathrm{dec}}^{-1}$, globally (WD) and north of 30$^{\circ }$N (NH). The map was created by using python3-mpltoolkits.basemap (version 1.2.1, https://matplotlib.org/basemap/).

Applying the WPI metric to ERA5 reanalysis data we see upward long-term trends of WPI in NH summer (Jun–Aug) from 1981 to 2019 (Fig. 1). The strongest signal of more than $+0.6\,{\mathrm{dec}}^{-1}$ can be found in a curved band stretching from North Atlantic, Southern Europe to Siberia, including highly populated areas of Central Europe. In other parts of the world the trends of WPI within this season are either weak or negative. In the NH (north $30^{\circ }$N) 19.1% of the land area has seen a positive trend in WPI stronger than $+0.6\,{\mathrm{dec}}^{-1}$ in contrast to 8.9% of land areas on global average. Remarkably, this percentage increases to more than 70% over Europe. The band is connecting the North Atlantic (the Atlantic Meridional Overturning Circulation, AMOC center) to other regions and therefore almost everywhere can be affected via the teleconnections, for example Mediterranean areas, Central Asia or even East China. A second area of increasing WPI is visible in the Southern Hemisphere (SH) winter over the southern part of Africa (Angola and Namibia). However, in our study we concentrate on pattern located in the NH. In the supplementary material (S5) more seasonal patterns including positive and negative WPI trend directions derived from NCEP/NCAR reanalysis data^27^ are provided. No discrepancy to Fig. 1 can be found.

### Seasonal anomalies of WPI

Remarkable changes in WPI can also be seen during different seasons of single years. Prominent examples of strong seasonal anomalies are the summer 2010 (Russian heatwave)^38,49^ and the summer 2018 (European drought)^2,3,25^. Both cases are good examples where (1) dynamical processes played an important role in the emergence of climate extremes and (2) hydro-climatic extremes occurred simultaneously at far away regions across NH mid-latitudes^30^.

**Figure 2:**
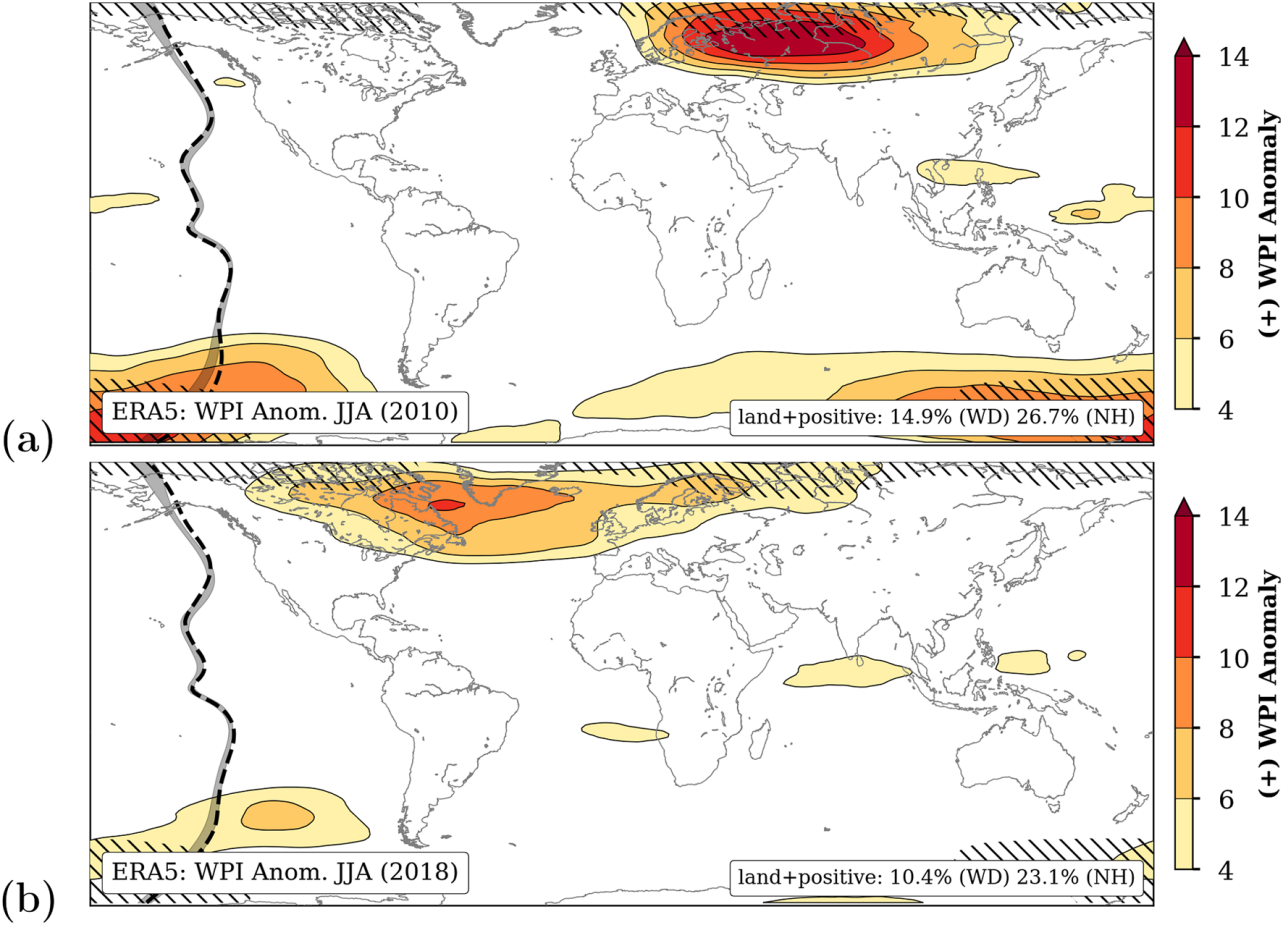
Observed anomalies of WPI pattern in Jun–Aug 2010 (**a**) and 2018 (**b**) compared to the long-term average 1981–2010 using ERA5 reanalysis data. Only positive anomalies are shown. The hatched areas are regions where the standard deviation of WPI is larger than 4. The values in the lower right box represent the percentage of grid cells over land showing positive anomalies larger than 4, globally (WD) and north of 30$^{\circ }$N (NH). The meridional distributions of the zonal mean WPI range ($\sigma$) for the climatological period (1981–2010) and the respective year are given as grey band and dashed line, respectively. The maps were created by using python3-mpltoolkits.basemap (version 1.2.1, https://matplotlib.org/basemap/).

Figure 2 shows the corresponding global patterns of seasonal WPI anomalies for 2010 Jun–Aug (Fig. 2a) and 2018 Jun–Aug (Fig. 2b) with respect to the long-term mean 1981–2010. Only positive anomalies associated with persistent large-scale circulation patterns are shown and the hatched areas mark regions where the standard deviation of WPI is larger than 4. This only concerns parts of the anomaly patterns at high-latitudes. In addition, the latitudinal distribution of the long-term zonal mean of the WPI (grey) and for the respective year (dashed) are shown on the left. Climatologically, a peak in WPI can be found in the subtropics at the edge of the Hadley cell at around 30$^{\circ }$N for NH summer. Yet, in the summers 2010 and 2018 strong positive anomalies exceeding the natural variability are evident at NH mid- and high-latitudes. The spatial maximum is located further north over Siberia largely overlapping in space and time with the Russian heat wave and record-breaking temperatures in Moscow. The hot and dry summer period over Europe in 2018 was similarly associated with northward shifted high WPI over a region extending from North America over the North Atlantic to Europe. The unusual period in Europe lasted the entire summer half of the year.

### Link between WPI and temperature and precipitation

The NH mid-latitudes have seen the strongest changes in WPI with increasing trends (Fig. 1) and events of unusual anomalies in summer (Fig. 2). We will thus focus on NH mid-latitude summer (30–70$^{\circ }$N) to quantify the correlation between summer means of WPI, temperature and precipitation data taken from the ERA5 global reanalysis data^20^.

**Figure 3:**
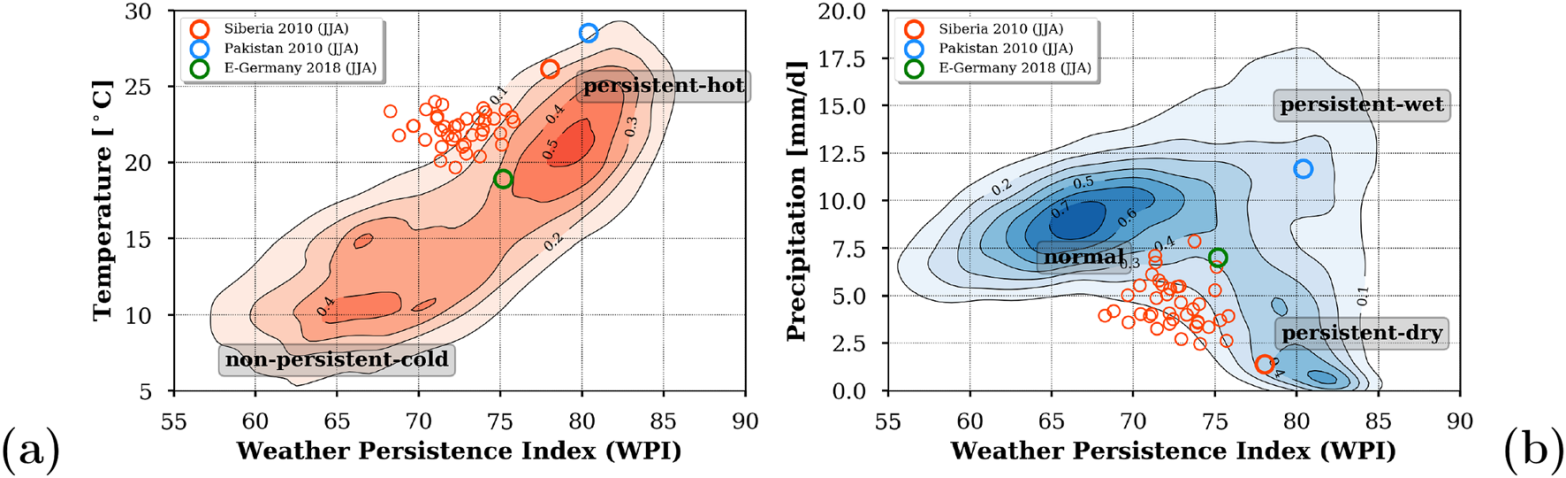
NH mid-latitude probability density patterns between summer means of WPI and temperature (**a**) and precipitation (**b**) from 1981 to 2019. The three circles correspond to conditions over Siberia (50$^{\circ }$N/50$^{\circ }$E, red), Pakistan (30$^{\circ }$N/70$^{\circ }$E, blue) in summer 2010 and Eastern Germany (52$^{\circ }$N/12$^{\circ }$E, green). Additionally, the individual years for Siberia (small red circles) are given for comparison to 2010. The plots were created by using python3-matplotlib (version 3.1.2, https://matplotlib.org/).

Figure 3 shows the 2-dimensional probability density function (PDF) patterns between WPI and temperature (Fig. 3a) and precipitation (Fig. 3b). The underlying values are annual ones of the summer means from 1981 to 2019 for all grid cells within 30$^{\circ }$N and 70$^{\circ }$N. A linear correlation is found between WPI and temperature (Fig. 3a)—the greater the WPI, the higher the temperature in summer. The exceptional situation of high WPI and high temperatures in 2010 shifted Siberia to the upper right corner of the distribution (red circle in Fig. 3a). The upper right part of the distribution is typically determined by subtropical regions at lower latitudes which are characterized by generally warmer and more persistent weather.

The precipitation pattern in Fig. 3b is more complex and contains 3 essential features: (1) normal conditions (2) persistent and wet conditions and (3) persistent and dry conditions. High persistence at NH mid-latitudes can hence locally favor either dry or humid conditions in addition to high temperatures. This confirms the hypothesis that both droughts and floods will become more likely in more persistent large-scale wind systems, depending on the relative position with respect to the northward or southward flow of the atmospheric waves. For instance, during the extreme NH summer in 2010 Siberia experienced high weather persistence associated with dry conditions over Siberia (red circle) and wet conditions over Pakistan (blue circle). The persistent jet stream led to rather cold airflow southward to Pakistan where it collapsed with warm monsoon winds. In other years the WPI was clearly lower, the temperature colder and the averaged daily rainfall higher. For comparison of the 2 years we added a third green circle representing the conditions over the eastern part of Germany in 2018.

To summarize, in normal years the summers over Siberia (thin red circles) are much less persistent and consequently also less hot and dry than in 2010.

**Figure 4:**
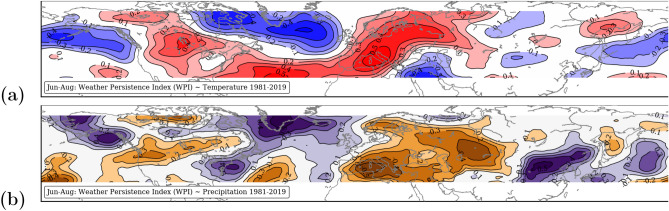
Correlation patterns between Jun–Aug means of weather persistence index (WPI) and temperature (**a**) and precipitation (**b**) from 1981 to 2019: positive (red, violet) and negative (blue, orange). The map were created by using python3-mpltoolkits.basemap (version 1.2.1, https://matplotlib.org/basemap/).

The NH correlation patterns between the summer means of WPI and temperature (a) and precipitation (b) from 1981 to 2019 are shown in Fig. 4. The red colored areas in Fig. 4a indicate positive correlations between WPI and temperature implying that summer with persistent weather conditions are associated with positive temperature anomalies. This is the case for most parts of Europe and Russia. A weak positive correlation is also evident over North-America. Over the NH ocean surfaces the correlation is negative. The interesting pattern is that the heat anomalies in Europe and North-America are temporally linked to a cold anomaly in the North-Atlantic. A possible connection to the AMOC weakening^4^ cannot be ruled out.

Figure 4b shows the results for precipitation. For most of the NH land masses, persistent weather patterns favor hot and dry summers. Areas of negative correlations are predominately over the oceans and along the coast lines as well as in the southeast of Central Asia. In Western and Central Europe, warm and persistent summers can be both dry and wet. Persistent summer conditions in Southern and Northern Europe are mostly dry and hot.

**Figure 5:**
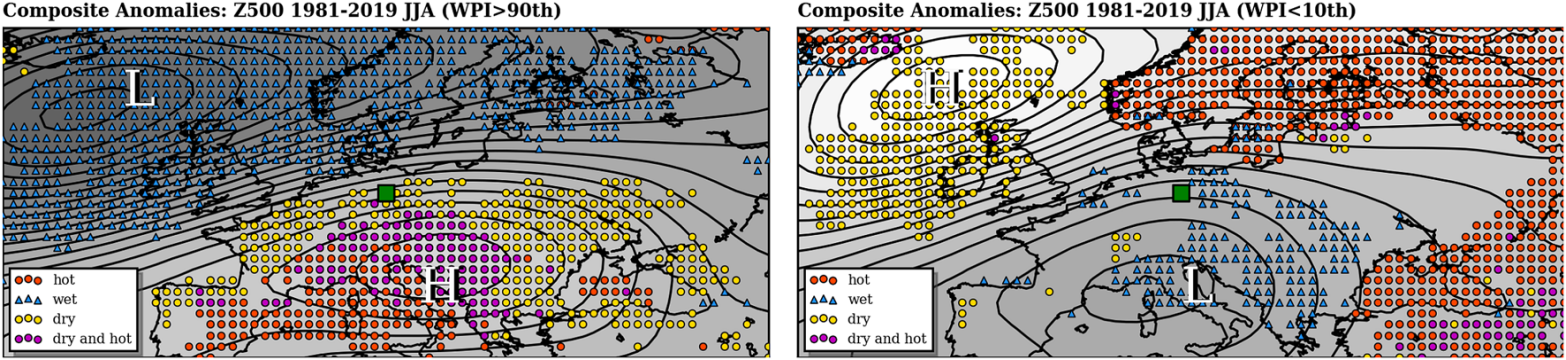
Composite patterns of Z500 anomalies from 1981 to 2019 related to 1981–2010 and Jun–Aug: WPI > 90th (**a**) and WPI < 10th (**b**) at the grid cell 52.0$^{\circ }$N/12.0$^{\circ }$E (green square) over Central Europe. Also added are hot anomalies (red, $+1.5$ K), wet anomalies (blue, $+1.5$ mm/d), dry anomalies (yellow, $-1.5$ mm/d) and both hot-dry (magenta). The maps were created by using python3-mpltoolkits.basemap (version 1.2.1, https://matplotlib.org/basemap/).

To analyze the prevailing circulation patterns during periods of high and low WPI over Central Europe in Jun–Aug composite patterns of Z500 anomalies were calculated for WPI > 90th percentile (Fig. 5a) and WPI < 10th percentile (Fig.5b), respectively. High persistence over Central Europe is associated with negative Z500 anomalies (contour lines) over Island and positive Z500 anomalies over South-East Europe. Such circulation patterns favor heat (red) and drought conditions (yellow) in Southern, Eastern and Central Europe. Wet conditions (blue) are mainly blocked from the continent and persist over the North-Atlantic with extensions to the British Islands and Scandinavia. The atmospheric circulation pattern during periods of low WPI (Fig. 5b) is reversed with positive Z500 anomalies over Island and negative anomalies over the Mediterranean Sea. The center of rainfall is located over most parts of Eastern Europe. Heat and drought conditions can be found over the North-Atlantic and Scandinavia. Since the WPI is defined for each region, similar considerations as shown here can also be carried out for other regions.

### WPI and hydro-climatic extreme events

**Figure 6:**
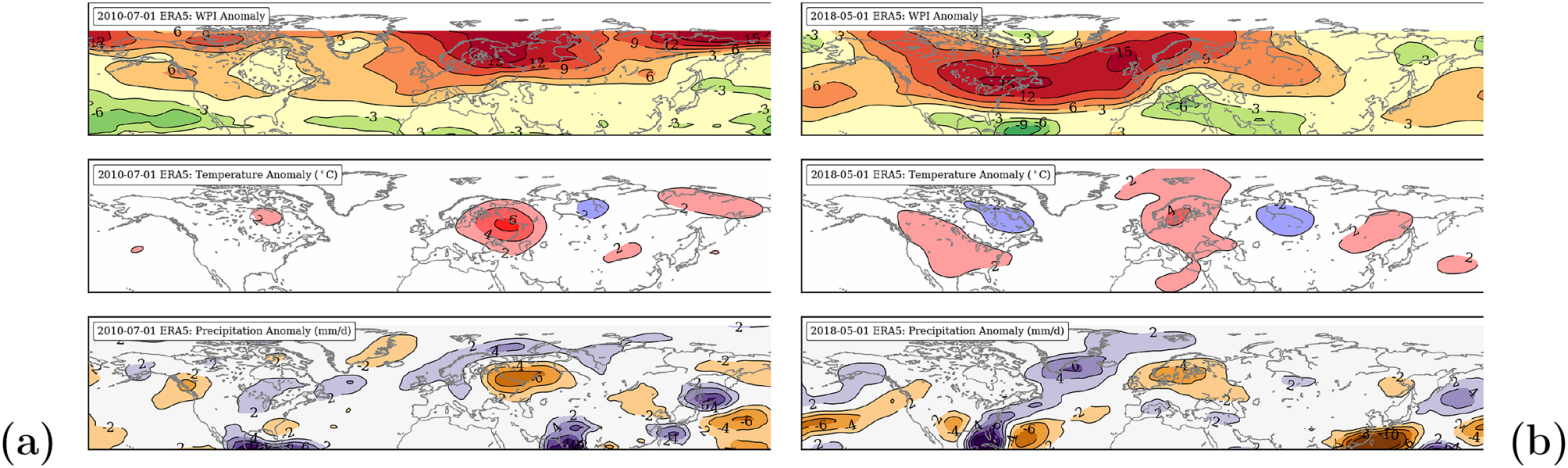
Observed patterns of WPI (top), temperature (center) and precipitation (bottom) anomalies averaged for July 2010 (**a**) and May 2018 (**b**) related to 1981–2010. The maps were created by using python3-mpltoolkits.basemap (version 1.2.1, https://matplotlib.org/basemap/).

Here we take a deeper look at the relationship of WPI and temperature and precipitation anomalies during the period of observed heat extremes in summer 2010 over Russia^32,38,49^ and in summer 2018 over Europe^3,25^ by using different forms of graphical representation. Figure 6 shows monthly anomaly patterns across the NH for July 2010 (Fig. 6a) and May 2018 (Fig. 6b). In July 2010, high WPI anomalies over Siberia led to heat and drought conditions over Russia. Simultaneously, permanent rainfall occurred over Pakistan leading to an historical flood event. Both extremes were linked by an omega like circulation pattern^28^ with the center over the western part of Russia. In May 2018, high WPI anomalies over the North-Atlantic and Europe led to the hottest May on record in Central Europe. This was the beginning of severe and persisting drought conditions in Europe.

**Figure 7:**
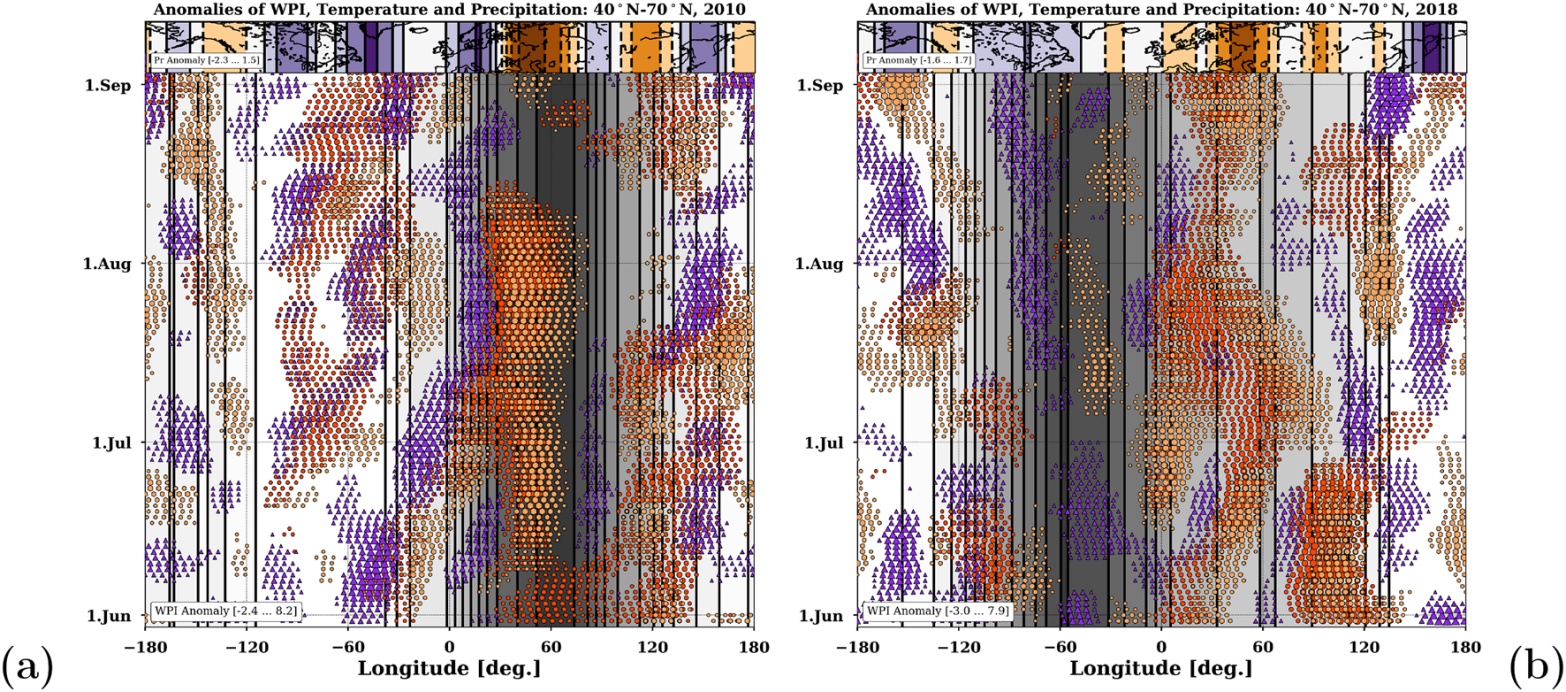
Hovmöller diagrams of positive WPI anomalies (grey contours) averaged over 40–70$^{\circ }$N from Jun–Aug 2010 (**a**) and 2018 (**b**). Overlaid are temporal evolution of longitudinal conditions of heat (red), wetness (purple) and drought (orange). The map (on top) shows longitudinal stripes of the total precipitation anomalies with regard to 1981–2010: purple (wet) and brown (dry). The reference period for all anomalies is 1981–2010 and the value ranges of the contours are given in the lower left boxes, respectively. The maps were created by using python3-mpltoolkits.basemap (version 1.2.1, https://matplotlib.org/basemap/).

A quasi temporally evolution of the two summer seasons 2010 and 2018 is shown in Fig. 7a and b. The Hovemöller diagrams contain grey stripes of the summer mean WPI anomalies and scatter points showing the longitudinal temperature (red) and precipitation (orange/purple) anomalies averaged over 40–70$^{\circ }$N. The stripped map on the top illustrates the longitudinal distribution of the summer mean precipitation anomalies, respectively. While in summer 2010 the peak of WPI anomaly is located around 60$^{\circ }$E, in summer 2018 the peak is located around 60$^{\circ }$W. The value range of WPI is given in the lower left box. Both cases show, that the location of the WPI maximum also determines the location of wet and dry anomalies. On the western side occur wet anomalies and around the center heat and droughts, mostly dominated by anticyclonic circulation patterns. Low values of WPI favor the alternation of wet and dry weather conditions and reduce the tendency for persisting hydro-climatic extremes.

### Ability of global climate models to reproduce weather persistence

We finally investigate the ability of selected CMIP5^48^ ensemble members to reproduce observed climatology and trends of weather persistence in climate simulations for the time period 1981–2019. Since the historical runs end in 2005 we extended the time period using 2006–2019 from the high-emission scenario RCP8.5. Five GCMs were selected for this purpose (see Table 1) which were part of prominent climate impact projects and initiatives such as the Inter-Sectoral Impact Model Intercomparison Project (ISIMIP)^19^ or the Coordinated Regional Downscaling Experiment (Cordex) for Europe^26^.

**Table 1:** Summary of the selected CMIP5 models used to trend analysis of WPI: percentage of land area showing positive trends of WPI in June–August of more than $+0.6\,{\mathrm{dec}}^{-1}$ (see Fig. 9): globally (WD) and only above $30^{\circ }\hbox{N}$ (NH).

Long name	Realization	Short name	Color	Time period	WD (%)	NH (%)
ERA5	Reanalysis	era5	Black	1981–2019	8.9	19.1
MPI-M-MPI-ESM-LR	r1i1p1	mpi	Red	1981–2019	18.3	42.3
IPSL-IPSL-CM5A-MR	r1i1p1	ips	Yellow	1981–2019	2.5	3.7
NCC-NorESM1-M	r1i1p1	nor	Magenta	1981–2019	4.9	0.7
CNRM-CERFACS-CNRM-CM5	r1i1p1	cnr	Green	1981–2019	0.5	0.9
MOHC-HadGEM2-ES	r1i1p1	had	Blue	1981–2019	5.2	8.1

**Figure 8:**
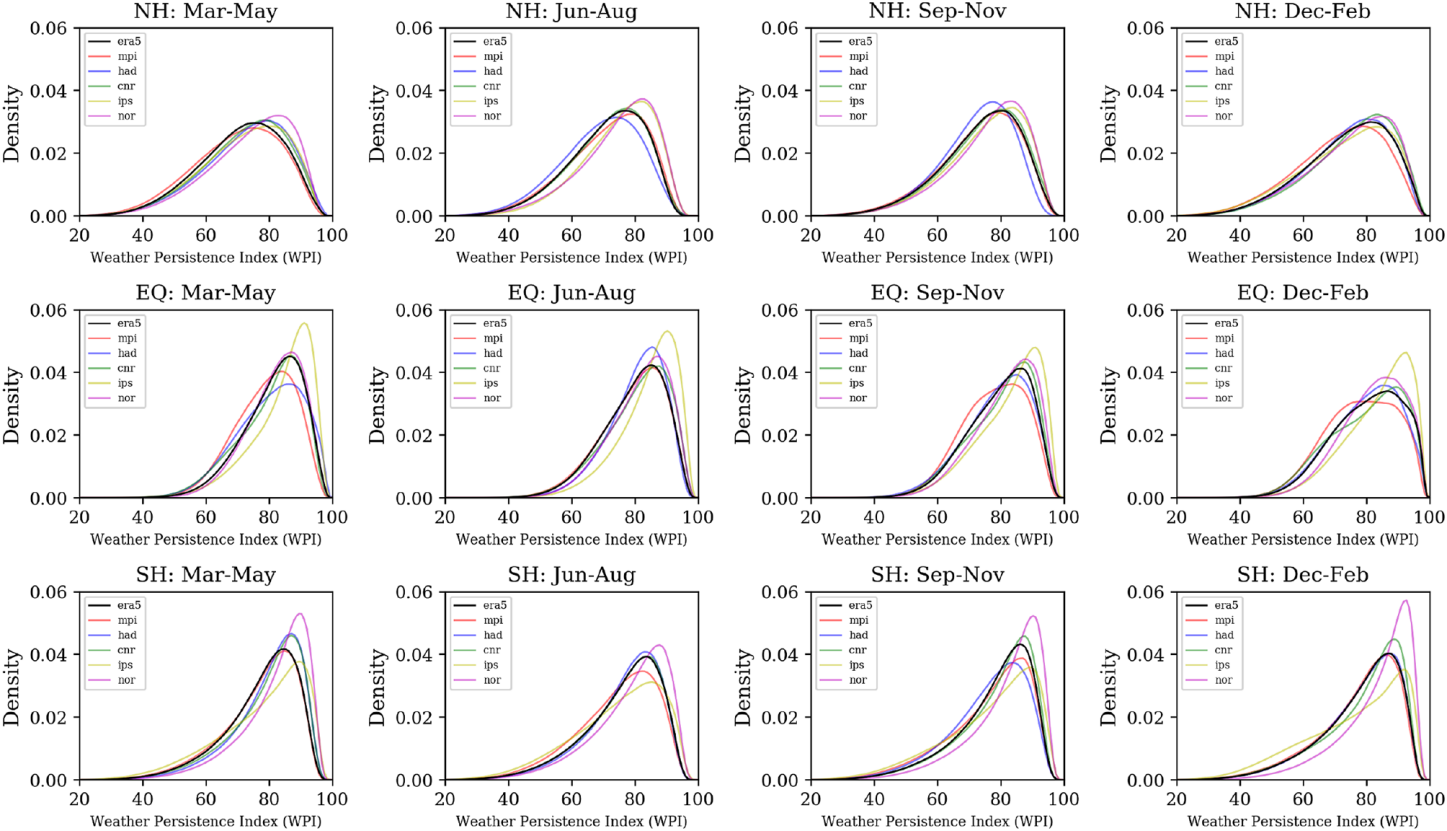
1981–2019 probability density functions of the weather persistent index (WPI): seasonal (columns), hemisphere (rows), ERA5 (black line) and CMIP5 (color lines). The following abbreviations stand for: NH (75–30$^{\circ }$N), EQ (30–30$^{\circ }$S) and SH (30–75$^{\circ }$S). The plots were created by using python3-matplotlib (version 3.1.2, https://matplotlib.org/).

Figure 8 shows an intercomparison of WPI for the time period 1981–2019. The derived probability density functions are grouped by seasons (columns) and hemispheres (rows). The WPI derived from ERA5 reanalysis data (era) is given as black lines and from the CMIP5 members (MPI-ESM-MR (mpi), IPSL-CM5A-LR (ips), NorESM1-M (nor), CNRM-CM5 (cnr) and HadGEM2-ES (had)) in different colors. The shapes of the respective curves are positive skewed with a maximum near $\hbox{WPI}=80$. In the NH, simulations of the GCMs are in good agreement with observations. Near the Equator and in the Southern Hemisphere GCMs still manage to reproduce the general shape of the distribution albeit with more variability in the position and intensity of the maximum. Overall, it can be concluded that GCMs are generally able to reproduce the averaged persistence of subsequent daily weather patterns.

**Figure 9:**
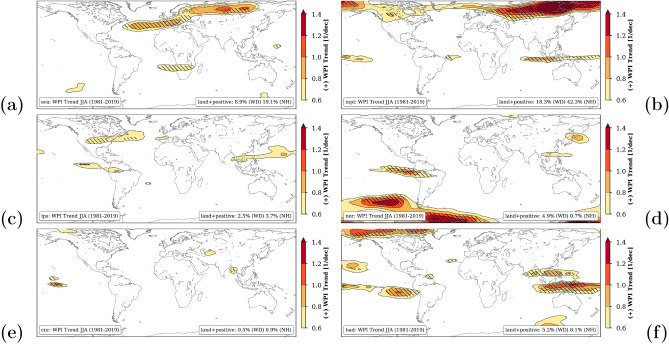
Global trend patterns of the weather persistence index (WPI) in Jun–Aug from 1981 to 2019 processed from ERA5 reanalysis data and selected CMIP5 simulations: ERA5 (**a**), MPI-ESM-MR (**b**), IPSL-CM5A-MR (**c**) and NorESM1-M (**d**) CNRM-CM5 (**e**) HadGEM2-ES (**f**). Only positive and significant trends (*p*-value < 0.05 using a two-sided *t*-test) are shown as contours and hatched, respectively. The values in the lower right box represent the percentage of grid cells over land showing positive trends larger than $+0.6\,{\mathrm{dec}}^{-1}$, globally (WD) and $>30^{\circ }$N (NH). The maps were created by using python3-mpltoolkits.basemap (version 1.2.1, https://matplotlib.org/basemap/).

The picture changes when analyzing the WPI trends. Basically, CMIP5 simulations are free simulations without data assimilation. Thereby, the timing of events are totally lost or even missing and thus influences trends in extremes. Figure 9 shows the trend patterns in Jun–Aug when applying WPI for ERA5 (a) and the five selected CMIP5 models and the period 1981–2019. The results are further summarized in Table 1 and indicate the percentage of land area affected by positive trends of WPI. Noticeable differences to observed results (Fig. 9a) are visible with disagreements in most parts of the world. Although similar structures are emerging for the MPI-ESM-MR simulation (Fig. 9b) in the Northern Hemisphere, there are remarkable differences evident over Europe with the band of increasing weather persistence shifted to the north implying that associated hydro-climatic extremes could be underestimated over large areas of Europe^41^. In general, the selected CMIP5 runs hardly show any systematical increase in weather persistence for the NH summer over Europe. There are first studies comparing atmosphere circulation patterns over Europe in CMIP6 with CMIP5 and they found an improvement in the new generation of climate model^5,14^. Nevertheless, they also recommend a careful selection of driving models for downscaling experiments.

The missing ability of the selected GCMs to reproduce trends in weather persistence cannot easily be corrected through statistical adjustments of distributions^19,22,34^. Therefore, it can be assumed that extreme hydro-climatic events and their sectoral impacts, which are largely caused by the duration of weather patterns, are underestimated in climate simulations^41^.

Supporting analyses in order to clarify the following questions are given in the supplementary material: (S1) How robust is the observed WPI trend pattern in summer (Fig. 1) when removing the year 2010? (S2) What explains the features in the PDF pattern in Fig. 3a? (S3) How change the PDF patterns (Fig. 3) for the individual years 2010 and 2018? (S4) Are there WPI anomalies with the magnitude of the 2010 event in the five selected CMIP5 models? (S5) What do the WPI trend patterns look like in the other seasons?

To summarize, the latter indicates that the WPI trends in boreal summer are more concise than in other seasons and most of the selected CMIP5 models underestimate events of high weather persistence especially over Siberia. The most similar pattern compared to ERA5 can be seen for the NorESM1-M simulation (nor). Even if the observed long-term trend from 1981 to 2019 (Jun–Aug) is calculated without 2010, the general shape of the pattern is retained.

## Conclusions

The systematic screening of atmospheric fields applying established methods of image recognition^50^ makes it possible to investigate and quantify the persistence of large-scale circulation patterns worldwide and to evaluate them using objective criteria. The structural image comparison method resembles the eye of a knowledgeable observer, who perceives structural differences and similarities of two images and rates them on a scale from $-1$ to 1. The findings contribute to a better understanding of the causes of extreme weather conditions and possible future developments, because the large-scale wind fields ultimately determine whether a heat wave, a flood or a drought can develop.

Our results illustrate that weather persistence in boreal summer on NH mid-latitudes has significantly increased since 1981 with an unusual strong anomaly seen in the summers of 2010 and 2018. The increase in number of persistent weather situations is a prominent evidence for changes in the atmospheric circulation likely favoring prolonged heat extremes^8^. The causal mechanism behind is the slow-down of the summer circulation in NH mid-latitudes and the quasi-resonant amplification of planetary waves^30,39^. Hoffmann^21,23^ recently showed that this phenomenon very likely leads to new dominant weather patterns over Europe and that about 20% of the observed temperature rise in Europe since 1990 is caused by changes in atmosphere circulation.

The developed WPI can serve as a predictor for hydro-climatic extremes. Thereby, every individual circulation pattern or location in the world is assigned an additional measure of weather persistence. The cause and the effect of weather extremes can thus be viewed more in context. High values in summer e.g. over Europe are strongly associated with positive temperature anomalies and a rain deficit. This often leads to heat waves and strong droughts. Also specific circulation patterns can be identified according to the magnitude of WPI. For Central Europe these are mostly zonally extended anticyclonic circulation pattern over Southern Europe while low air pressure prevails over Iceland. The more northerly high values of WPI occurs, the more Central Europe is affected by heat waves and droughts. In times with low persistence, the patterns are often reversed. Intensive and permanent rainfalls occur as a consequence of moisture transport from the Mediterranean into Central Europe.

The summer half of 2018 was particularly unusual in terms of long-lasting weather patterns over the NH mid-latitudes. From April to September, strong positive WPI anomalies dominated over the North Atlantic, resulting in extreme drought and heat conditions in most regions of Europe^3,25,30^. Already in 2010, persistent weather patterns led to a historical heat wave over Russia and, at the same time, to severe flooding in Pakistan^32^. Undeniable, the significant trends towards higher persistence in the NH summer (Fig. 1) increase the likelihood for hydro-climatic extremes (Fig. 4).

Basically, the WPI method provides a global picture of weather persistence. The trends to longer persistence are remarkable over Europe and Siberia and in the boreal summer. These are also the regions, where recent extremes have been associated with the occurrence of resonant planetary waves^29,39^. However, a more comprehensive evaluation of regional patterns and links to weather persistence and hydro-climatic extremes is needed and recommended.

The subsequent question, whether simulated atmospheric fields of GCMs show similar tendencies in persistence of weather patterns was investigated using output of five GCMs. The results show that on average the WPI distributions are comparable, but there were remarkable deviations in the historical trends. While the WPI values across Europe are increasing when analyzing reanalysis data, the climate model simulations show for the same period of time a rather uniform underestimation. Because weather persistence has a decisive influence on the frequency and intensity of extreme hydro-climatic events^35^, the consequence and peril is that scenario data from global and hence, very likely also regional climate models (RCMs), cannot yet adequately reflect these extremes and also not related trends and impacts.

## Methods

The state and variability of the atmosphere circulation consists of various time-dependent spatial patterns. Due to their unlimited variety, they are hard to identify in daily reanalysis fields or climate simulation data. The same applies to their synoptic interpretation. Patterns emerge and disappear, relocate or stay in place. While the relocation properties of patterns require more complex approaches, the measure of duration and persistence of the prevailing pattern is more promising to analyze. For each location and day we bring together the cause and effect of weather. No matter which circulation pattern prevails over a region, it gets a value for the persistence tendency. Other methods tend to look for e.g. familiar blocking patterns at a specific region^6,14,40^. Thus, our methodology is based on the routine work of meteorologists in the forecast service, who have to evaluate structural developments in weather forecast map. Thereby, we grasp weather maps as images that can be compared to each other.

**Figure 10:**
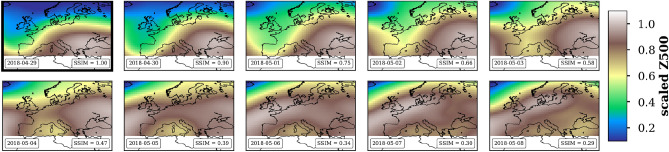
Illustration of the Structural SIMilarity (SSIM) image comparison approach applied to 10 successive atmospheric circulation patterns (scaled Z500) over Europe from 2018–04–29 (reference image, upper left) to 2018–05–08 (lower right). The reference image shows a trough over Western Europe. The SSIM values are given in the lower right box, respectively. The maps were created by using python3-mpltoolkits.basemap (version 1.2.1, https://matplotlib.org/basemap/).

For this purpose we applied a Structural SIMilarity (SSIM) image comparison approach^1,50^ in order to identify similarities of 10 successive atmospheric images for a domain with a size of $\Delta \,\hbox{lat}=30^{\circ }$ (latitude) and $\Delta \,\hbox{lon}=70^{\circ }$ (longitude) as illustrated in Fig. 10. The spatial expansion is based on the synoptic scales of meteorological phenomena. We used the code of the metric available within the python library *scikit-image* (version: skimage 0.18). The underlying daily atmospheric field is the geopotential height at 500 hPa (Z500) taken from ERA5^20^. It represents and includes the most relevant dynamical features of the middle troposphere. Mid-latitude cyclonic and anticyclonic weather patterns near surface are embedded in wave-like circulation patterns, *trough* and *ridge*, respectively. In addition, a 3-days moving average is applied for smoothing. By shifting the analysis window in space ($1^{\circ }$) and time (1d) we obtain a nearly global picture of weather persistence (from $75^{\circ }$S to $75^{\circ }$N). Consequently, we call the new measure weather persistence index (WPI). Existing connections beyond the synoptic scale are also recorded and detected.

### From the structural SIMilarity to the weather persistence index

The novel way to detect and quantify weather persistence globally is the setting in which the image comparison method is embedded. The used Structural SIMilarity (SSIM) metric is one of several approaches^36^. The starting point is the raw data (Z500) of two subsequent images $\mathbf {x}$ and $\mathbf {y}$ having a size of $70\times 30$ pixels. The two images are locally compared by calculating $\mathrm{SSIM_{j}}$ for a moving window of $7\times 7$ pixels (j) considering three independent variables: luminance (l), contrast (c) and structure (s). The combination of the three components results in the following equation of the similarity metric:

1 $$\begin{aligned} {\textit{SSIM}}_{j}(\varvec{\mathbf {x}},\mathbf {y})= \left( \dfrac{2\mu _{x}\mu _{y}+C_{1}}{\mu _{x}^{2}+\mu _{y}^{2}+C_{1}}\right) _{l}\cdot \left( \dfrac{2\sigma _{x}\sigma _{y}+C_{2}}{\sigma _{x}^{2}+\sigma _{y}^{2}+C_{2}}\right) _{c} \cdot \left( \dfrac{\sigma _{xy}+C_{3}}{\sigma _{x}\sigma _{y}+C_{3}}\right) _{s}= \dfrac{\left( 2\mu _{x}\mu _{y}+C_{1}\right) \cdot \left( 2\sigma _{xy}+C_{2}\right) }{\left( \mu _{x}^{2}+\mu _{y}^{2}+C_{1}\right) \cdot \left( \sigma _{x}^{2}+\sigma _{y}^{2}+C_{2}\right) } \end{aligned}$$

The term $\mu$ stand for the mean intensity (luminance), $\sigma$ for the standard deviation (contrast) and $\sigma _{xy}$ for the correlation (structure) of the two images, respectively. $C_{1}=0.01$ and $2C_{3}=C_{2}=0.03$ are constants for reasons of stability when the denominator is very small^50^. The SSIM measure of the entire image is computed by averaging over all local ${\textit{SSIM}}_{j}$ values. The resulting value of the SSIM index ranges from $-1$ (inverse) over $\ll 1$ (non-similarity) to 1 (identity). The SSIM index is calculated for the 10 consecutive atmospheric patterns (i) and then averaged. The first image serves as a reference. From that point on we call it weather persistence index (WPI): ${\textit{WPI}}=100\cdot \frac{1}{10}\sum _{i=1}^{10}{\textit{SSIM}}_{i}$, where 100 is a scaling factor and ${\textit{SSIM}}_{1}$ is the identity by self-comparing the reference image.

Basically, WPI is a suitable measure to detect and quantify the persistence of prevailing circulation patterns in any region of the world. The size of the domain (image) and the length of the temporal window are the degrees of freedom and were estimated using the spatial and temporal scale of hydro-climatic extremes. The following paragraph sketches the necessary data processing steps.

### Workflow

The steps to be carried out one after the other:

- *Step 1*: remapping of the daily Z500 global reanalysis fields using bi-linear interpolation ($\Delta =1^{\circ }$) and 3-day averaging
- *Step 2*: defining a $1^{\circ }$ moving spatial window ($\Delta \,\hbox{lat}=30^{\circ }$, $\Delta \,\hbox{lon}=70^{\circ }$) and a 1-day moving temporal window of 10-days to detect similarity of consecutive patterns after linear scaling from 0 to 1 (including the self comparison)
- *Step 3*: space–time screening of 10-day structural similarity (stepwise globally, from 1981 to 2019)
- *Step 4*: aggregation to seasonal means (e.g. JJA) and analyzing anomalies and long-term trend patterns globally (applying *T*-test for testing significance)
- *Step 5*: repeating step 1–4 to global temperature and precipitation fields without to calculate the SSIM for a smaller spatial window ($\Delta \,\hbox{lat}=10^{\circ }$, $\Delta \,\hbox{lon}=20^{\circ }$)

The outlined workflow was further applied to global climate simulations.

### Validation

The validation of the approach is done by using real atmosphere data as shown in Fig. 10. The upper left image is the reference one and represents the circulation conditions on the 29th of April 2018. It illustrates a wave like circulation pattern with a very pronounced structure of a trough over Western Europe. The other ones are subsequent patterns shifted by 1 day until the 8th of May. We see how the reference pattern changes over time. However, this example only serves as illustration. In the boxes of each figure the corresponding date and the value of the calculated SSIM are given. Consequently, the comparison of one and the same pattern results in $\hbox{SSIM}=1.0$. The comparison of the first pattern with the 9 consecutive patterns results in further SSIM values lower than 1 and finally the averaging over time the new weather persistence index (WPI). The higher the mean SSIM the higher the WPI. We have chosen the 10-day window because the typical time scale of synoptical systems at mid-latitudes is about 10 days. The first image comparison starts with the self comparison of the reference image (identity).

### Interpretation

**Figure 11:**
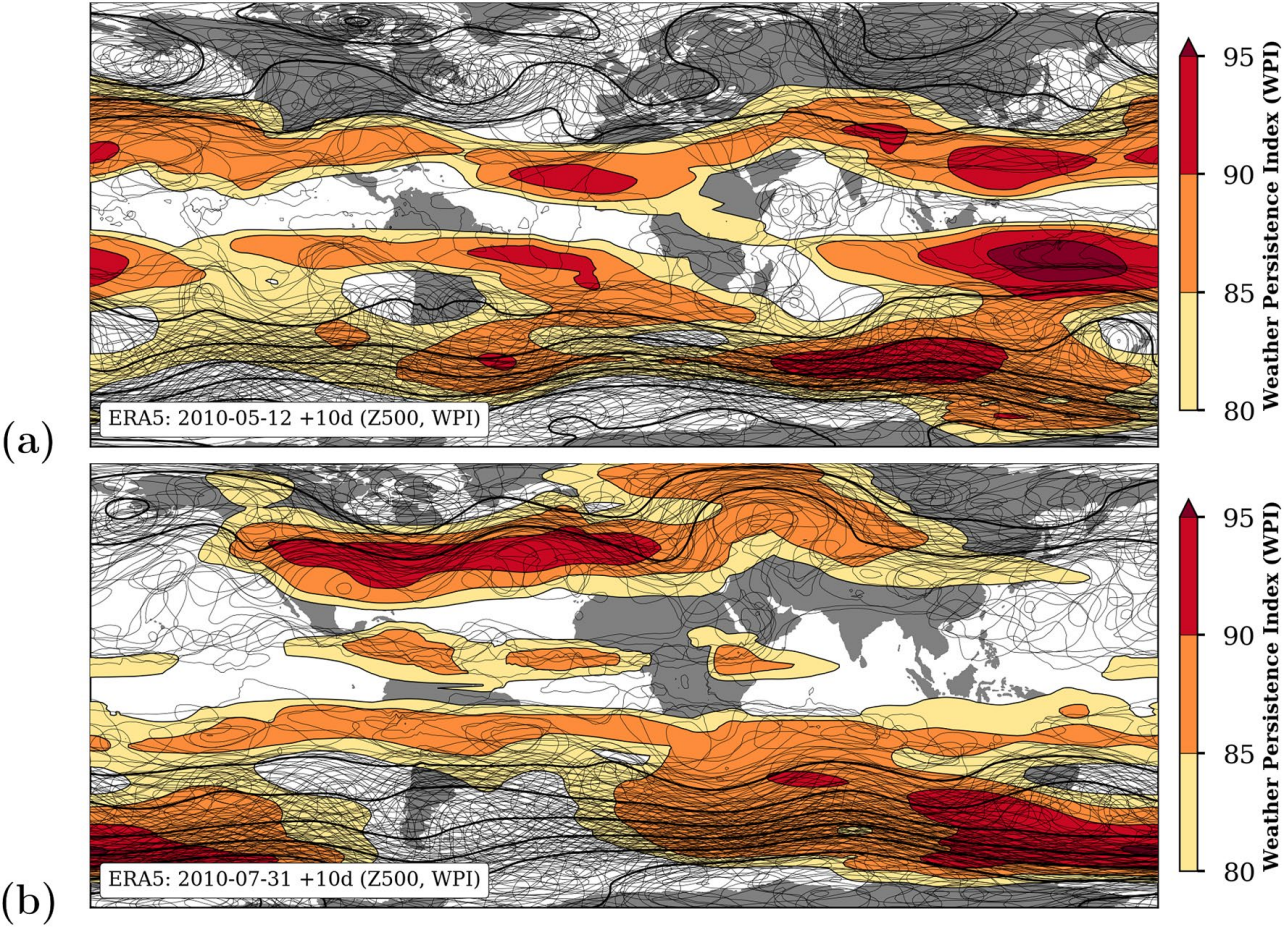
Global maps of Z500 contours of 10 consecutive days for two selected dates in 2010 representing high and low weather persistence over Europe: 2010–05–12 (**a**) and 2010–07–31 (**b**). The 10-day average of Z500 is given as thick solid line and the corresponding pattern of the WPI as shades contours. The spaghetti lines represent 10 individual daily Z500 contour lines within a given interval from 45,000 to 60,000 gpm (thin lines). The maps were created by using python3-mpltoolkits.basemap (version 1.2.1, https://matplotlib.org/basemap/).

The weather persistence index (WPI) is introduced here as a new measure of the structural similarity (SSIM) of day-to-day atmosphere images over a certain geographical region. Figure 11 illustrates the relation between the order of Z500 isolines of 10 consecutive days (thin black solid lines) and the corresponding WPI (shaded contours) for two situations in 2010: the 12th May 2010 (Fig. 11a) and the 31st July 2010 (Fig. 11b). The two situations differ strongly across Europe and Siberia in terms of weather persistence. In July 2010 the successive Z500 isolines over the North-Atlantic, Europe and Siberia run in orderly lanes over days or even weeks indicated by a high level of WPI (Fig. 11b). In contrast to it, the trajectories of the Z500 isolines in May 2010 appear rather disordered (Fig. 11a) associated with low WPI values. The underlying metric for calculating WPI overlooks the similarity of successive weather patterns and returns a value describing the temporal evolution of synoptic weather events. This has a far-reaching relevance for the atmosphere diagnostic and prediction of hydro-climatic extremes, such as the Russian heat wave in July 2010.

### Software

For the pre-processing of all netcdf files are used CDO (version 1.7.0) and for the calculation, the statistical analysis and the plotting of WPI are used the following python3 packages: matplotlib, basemap, numpy, scipy and skimage (v0.18).

## Supplementary information

Supplementary Information.
