# Author Correction: Atmosphere similarity patterns in boreal summer show an increase of persistent weather conditions connected to hydro-climatic risks

**DOI:** 10.1038/s41598-021-03671-4

**Published:** 2021-12-14

**Authors:** Peter Hoffmann, Jascha Lehmann, Bijan Fallah, Fred F. Hattermann

**Affiliations:** Potsdam-Institute for Climate Impacts Research, Climate Resilience, 14412 Potsdam, Germany

Correction to: *Scientific Reports* https://doi.org/10.1038/s41598-021-01808-z, published online 24 November 2021

The original version of this Article contained an error in the spelling of the author Bijan Fallah which was incorrectly given as Bijan H. Fallah. The original Article and accompanying Supplementary Information file have been corrected.

