# Supplementary material for: Atmosphere similarity patterns in boreal summer show an increase of persistent weather conditions connected to hydro-climatic risks
Source: Sci Rep. 2021 Nov 24;11:22893. doi: 10.1038/s41598-021-01808-z (PMC8613183; doi:10.1038/s41598-021-01808-z)
Supplement: Supplementary file 1 — Supplementary Information. [file 41598_2021_1808_MOESM1_ESM.pdf]

Atmosphere similarity patterns in boreal summer show an increase of persistent weather conditions connected to hydro-climatic risks

Peter Hoffmann[*1], Jascha Lehmann[1], Bijan Fallah[1] and Fred F. Hattermann[1]

[1]Potsdam-Institute for Climate Impacts Research, Climate Resilience, Potsdam, 14412, Germany

Supplementary material

S1: Trend pattern excluding 2010 and variability

The magnitude of the trend pattern over Siberia is clearly reduced excluding 2010 (Fig.S1b) compared to all years (Fig.S1a). The magnitude of the pattern over Europe is nearly unchanged by the extreme summer 2010.

(a)

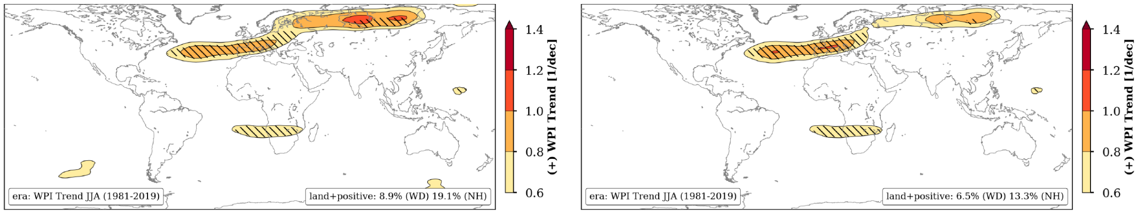

(b)

Figure S1: Long-term trend patterns of WPI in summer from 1981 to 2019 with 2010 (a) and without 2010 (b). [The maps were created by using python3-mpltoolkits.basemap (version 1.2.1, https://matplotlib.org/basemap/)].

The interannual variability of the summer mean WPI over NH midlatitudes from 1981-2019 is shown in Figure S2 by using cumulated anomalies related to 1981-2000. The courses rise/decline if positive/negative anomalies dominate. Both are evident and form a funnel after 2000. Europe (EU, dashed line) is located outside of the upper branch of the funnel (75th percentile) with increasing WPI anomalies. It also shows a cyclical variation with maxima around the extreme summers in NH 2003, 2010 and 2018.

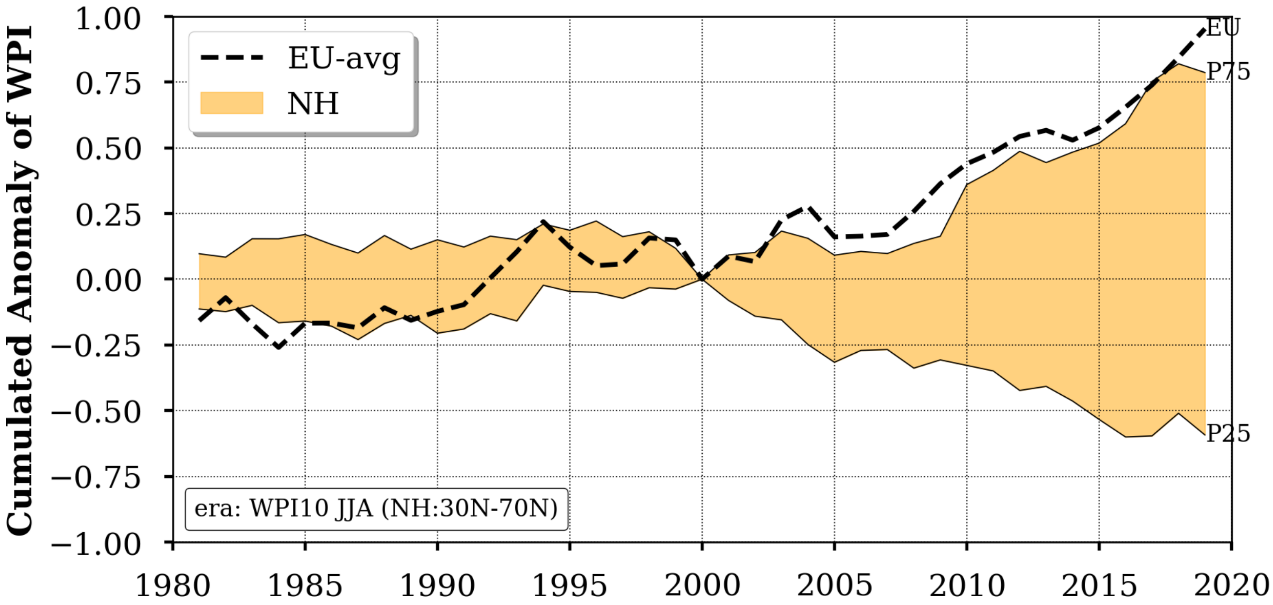

Figure S2: The temporal evolution is given as cumulated WPI anomalies over NH midlatitudes (30°N-70°N) from 1981-2019 related to 1981-2000. The upper and lower enveloping lines represent the 25th and 75th percentile of the grid cells. The mean values for Europe are given as a dashed line. [The plot was created by using python3-matplotlib (version 3.1.2, https://matplotlib.org/)]

S2: Secondary maximum

The two features within the PDF (Fig.S3a) result in from the land-ocean distribution over the NH mid-latitudes. The lower maximum on the cold end of the PDF is located over the North-Pacific. The other one in the upper right represents the mean continental areas. The long-term mean pattern of WPI in summer (Fig.S3b) shows a much stronger meridional gradient over the NH continents (North-America and Eurasia) than over the North-Pacific. The conditions there are less persistent and colder.

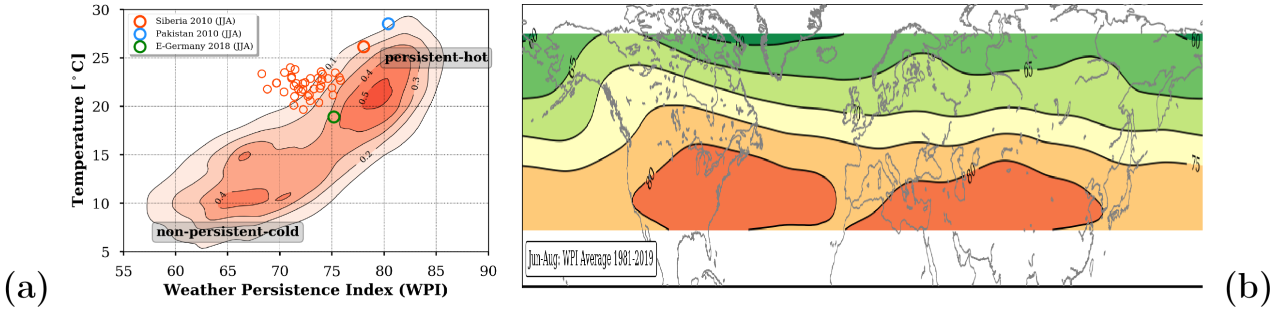

Figure S3: NH mid-latitude probability density patterns between summer means of WPI and temperature from 1981 to 2019 (a) – the same as Fig.3a. The long-term mean pattern of WPI in summer over the NH mid-latitudes (b). [The map was created by using python3-mpltoolkits.basemap (version 1.2.1, https://matplotlib.org/basemap/)].

S3: PDFs for individual years

Figure S4 shows the PDFs analog to Fig.3, however, for the individual years 2010 (top) and 2018 (bottom). The main features are similar. In the summers 2010 and also 2018 against it persisting weather conditions

in summer were stronger associated with hot and dry conditions compared the normal state shown in Fig.3.

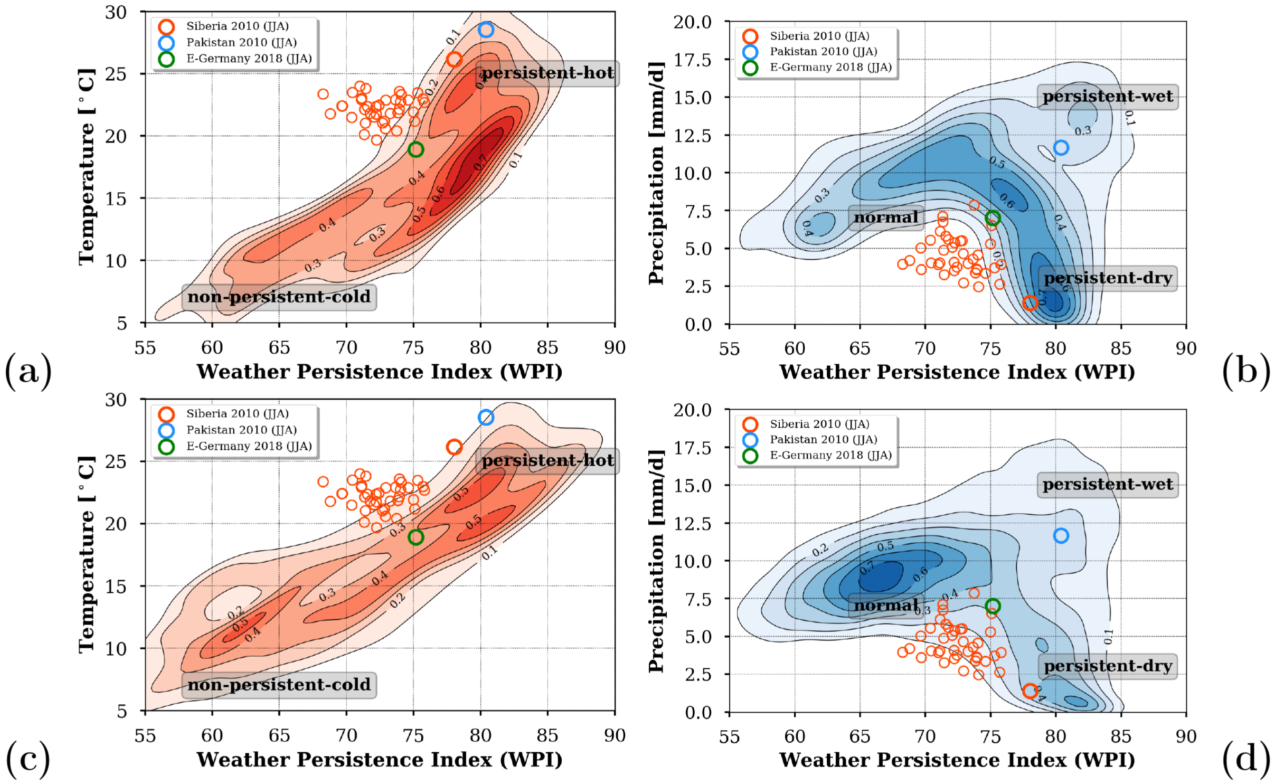

Figure S4: NH mid-latitude probability density patterns between summer means of WPI and temperature (a,c) and precipitation (b,c) from 1981 to 2019 for single years 2010 (a, b) and 2018 (c, d). [The plots were created by using python3-matplotlib (version 3.1.2, https://matplotlib.org/)].

S4: Do any of the selected CMIP5 model produce the magnitude of WPI over Russian in summer 2010?

Figure S5 show PDF patterns per GCM, where a critical magnitude of the WPI anomaly larger than 10 often occur and is in the order of the summer 2010 event over Siberia. While the analysis of the ERA5 reanalysis data shows high densities over Northern Europe and Siberia, the feature is much lower for the selected CMIP5 models, except for NorESM1-M (Fig.S5d). The locations of the maxima fit quite well to the ERA5 pattern (Fig.S5a). However, the length of 40 years is not sufficient for any fundamental conclusions or assessment of climate models. More systematic analyzes are required.

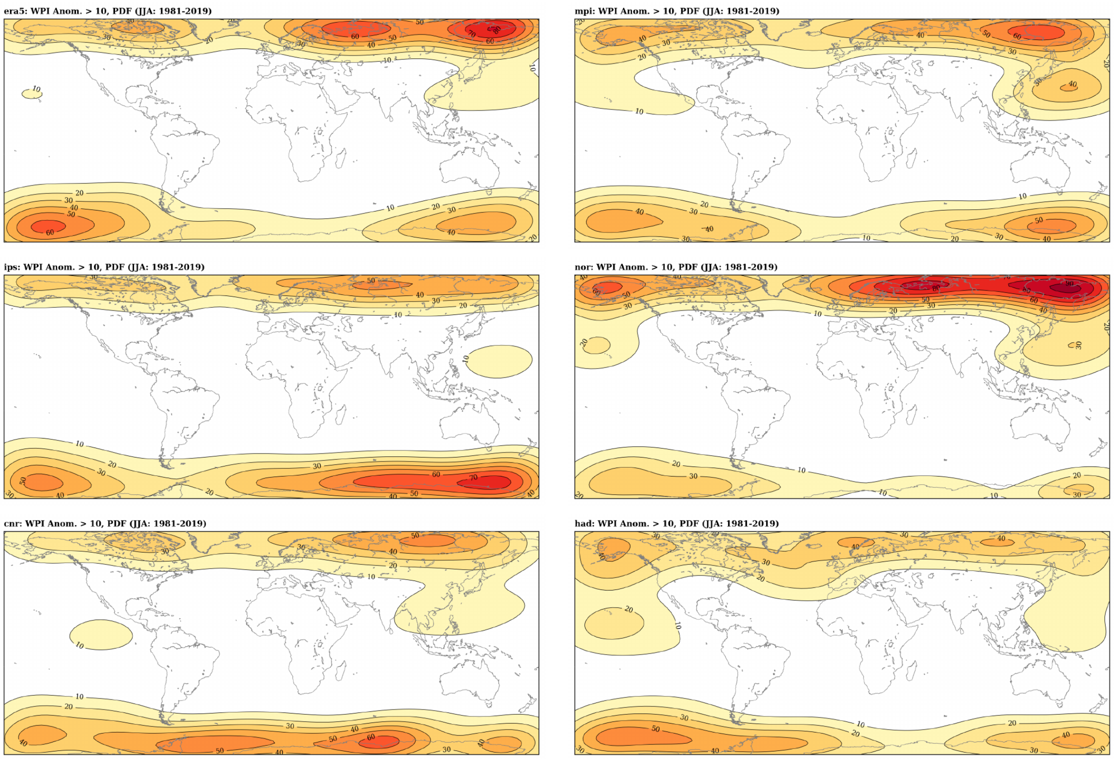

(a) (b) (c) (d) (e) (f)

Figure S5: Global maps showing the two dimensional probability density function of monthly mean WPI anomalies larger than 10 from 1981-2019 (Jun-Aug) regarding 1981-2010: ERA5 (a) and the 5 selected GCMs (b-f). [The maps were created by using python3-mpltoolkits.basemap (version 1.2.1, https://matplotlib.org/basemap/)].

S5: Long-term seasonal means and trends of WPI patterns using NCEP/NCAR reanalyses

The main part of the paper only discuss the conditions in boreal summer by using ERA5 reanalysis data. Here, we provide more additional materials across seasons and regions using NCEP/NCAR reanalysis from 1981 to 2020. Figure S6 collects global patterns showing seasonal characteristics of long-term means (left column) and long-term trends (right column). The trend pattern for June to August (Fig.S6f) is quite similar compared to the analyses based on ERA5 (Fig.S1a). However, another region showing increasing trends is visible over the Gulf of Alaska. No other season or region is stronger effected by weather persistence changes than the NH mid-latitudes, especially the North-Atlantic and Eurasia.

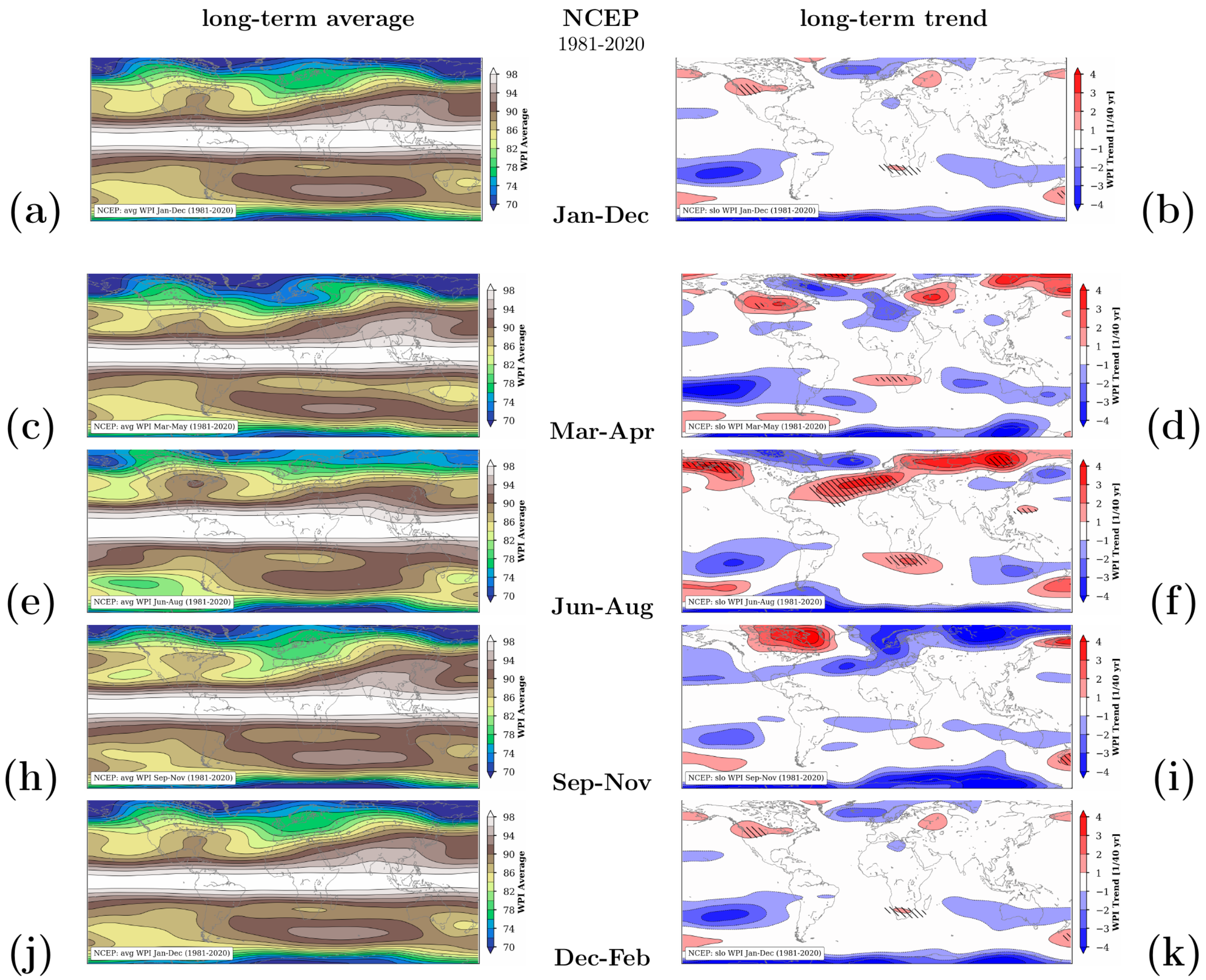

Figure S6: Long-term seasonal means and trends of WPI patterns derived from NCEP/NCAR reanalysis from 1981-2020. The hatched areas highlight regions, where the trend values are larger than the standard deviation. [The maps were created by using python3-mpltoolkits.basemap (version 1.2.1, https://matplotlib.org/basemap/)].
